# Lactate Inhibits the Pro-Inflammatory Response and Metabolic Reprogramming in Murine Macrophages in a GPR81-Independent Manner

**DOI:** 10.1371/journal.pone.0163694

**Published:** 2016-11-15

**Authors:** Agustina Errea, Delphine Cayet, Philippe Marchetti, Cong Tang, Jerome Kluza, Stefan Offermanns, Jean-Claude Sirard, Martin Rumbo

**Affiliations:** 1 Instituto de Estudios Inmunológicos y Fisiopatológicos–CONICET- Nacional Universtity of La Plata, 1900, La Plata, Argentina; 2 Institut Pasteur de Lille, Inserm, CNRS, Univ. Lille, CHU Lille, U1019—UMR8204—CIIL—Center for Infection and Immunity of Lille, F-59000, Lille, France; 3 Univ. Lille, Inserm, CHU Lille, UMR-S 1172—JPArc—Centre de Recherche Jean-Pierre AUBERT Neurosciences et Cancer, F-59000, Lille, France; 4 Department of Pharmacology, Max Planck Institute for Heart and Lung Research, D-61231, Bad Nauheim, Germany; 5 Medical Faculty, J.W. Goethe University, D-60590, Frankfurt, Germany; Institut National de la Santeet de la Recherche Medicale (INSERM), FRANCE

## Abstract

Lactate is an essential component of carbon metabolism in mammals. Recently, lactate was shown to signal through the G protein coupled receptor 81 (GPR81) and to thus modulate inflammatory processes. This study demonstrates that lactate inhibits pro-inflammatory signaling in a GPR81-independent fashion. While lipopolysaccharide (LPS) triggered expression of IL-6 and IL-12 p40, and CD40 in bone marrow-derived macrophages, lactate was able to abrogate these responses in a dose dependent manner in *Gpr81*^*-/-*^ cells as well as in wild type cells. Macrophage activation was impaired when glycolysis was blocked by chemical inhibitors. Remarkably, lactate was found to inhibit LPS-induced glycolysis in wild type as well as in *Gpr81*^*-/-*^ cells. In conclusion, our study suggests that lactate can induce GPR81-independent metabolic changes that modulate macrophage pro-inflammatory activation.

## Introduction

Low-molecular-weight metabolites such as bacterium-derived short chain fatty acids (SCFAs) have emerged as important local modulators of immune cell function[1, 2]. Among fermentation end products, lactate is the only one of these metabolites produced by both (i) lactic acid bacteria (i.e. *Lactobacillus* and *Lactococcus* species) and other intestinal microorganisms during the fermentation of carbohydrates, and (ii) the host metabolism at homeostasis and during disease states, such as infection and cancer. Consequently, immune cells migrating to different body sites may be exposed to variable lactate levels in different microenvironments. The gastrointestinal mucosa is exposed transiently to high lactate concentration from fermented food in the proximal segments[3]. Thus, the lactobacilli, bifidobacteria and other microbiota members can produce10 mM in steady state in the terminal ileon or colon [4, 5]. However, differences in diet and microbial composition, as well as surgical or pharmacological interventions may increase the lactate intestinal levels [6]. Concentrations of lactate > 100 mM are found in microenvironments such as the vagina in which lactic acid bacteria are the most represented population [7]. Furthermore, in specific conditions, host cells can produce lactate, as exemplified during macrophage M1 activation during infection [8, 9]. Otherwise, proliferating cancer cells normally acquire a high glycolytic rate metabolism with low oxygen consumption, the so called “Warburg metabolism” that results in increased lactate concentration in the tumor environment [10].

There is evidence that lactic acid or lactate modulate the maturation of monocytes, macrophages and other myeloid cells contributing to non-inflammatory phenotypes [11–13]. The molecular mechanisms responsible for this activity are not clear, being the capacity to activate specific GPCR or histone deacetylases, possible mediators of such effects [3]. GPR81 (or HCAR1) is the only GPCR described so far with capacity to bind lactate in physiological concentrations [14, 15]. A recent study reported that the exogenous administration of lactate inhibits the Toll-like receptor 4 (TLR4)-dependent pro-inflammatory responses in murine and human macrophages stimulated by LPS [16]. Using siRNA, it was suggested that the inhibitory effect is dependent on GPR81 signaling and it may be active in *in vivo* pathological situations like toxic hepatitis and pancreatitis [16].

Taking into account that multiple situations may contribute to changes in lactate concentration in the cellular environment, it is important to elucidate how endogenous or exogenous lactate influences overall immune responses to characterize disease mechanisms and develop novel prophylaxis methods. Aiming at further determining the pathways associated with the modulatory capacity of lactate on innate immune cells, we here described the use of bone marrow-derived macrophages (BMM) from GPR81 genetically deficient mice. Our findings indicate that lactate blocks LPS activation of macrophages in a GPR81-independent manner by modulating cell metabolic activity.

## Material & Methods

### Reagents

Racemic DL-lactic acid (J. T. Baker, USA) and NaOH 1 mol/L was used to prepare an aqueous lactate solution at 0.5 mol/L, pH 7.4. Lactate solutions were then filtered through a 0.45 μm membrane filter and stored at -20°C. Ultrapure LPS from *Escherichia coli* serotype 0111:B4 was from InvivoGen (Toulouse, France). All cell culture media, serum, and supplements were purchased from Gibco®. Glycolysis inhibitors 2-deoxyglucose and 3-bromopyruvate were purchased from Sigma-Aldrich (Lyon, France)

### Animal procedures: Macrophage generation

Female C57BL/6J (6–8 weeks old) mice were obtained from Janvier Laboratories (St. Berthevin, France). *Gpr81*^*-/-*^ and wild type littermates were generated as previously described [17]. To perform the experiments involving *ex vivo* cell analysis, animals were euthanasied by cervical dislocation under xilazine/ketamine i.p. anesthesia (French protocol CEEA 052011). Animals were maintained at the animal facility of Institut Pasteur de Lille (agreement#A59-350009). All experiments complied with current national and institutional regulations and ethical guidelines and the procedure was approved by the Institutional Animal Care and Use Committee (IACUC). Bone marrow (BM) from femurs and tibias was used as a source of hematopoietic cell precursors to differentiate macrophages (BMM) as previously described [18]. Peritoneal macrophages were obtained by flushing the peritoneal cavity with 5 mL of PBS and adhesion for 2h at 37°C on culture plates. Cells were left untreated or stimulated with (i) 100 ng/mL of LPS, (ii) various concentrations of lactate, (iii) combinations of LPS and lactate, or (iv) combinations of LPS and glycolysis inhibitors. Cells were processed after 3h stimulation for gene expression analysis or after 16h stimulation for cytokine secretion or flow cytometry analysis. Cell numbers and viability were assessed using Trypan blue solution and Countess^TM^ Automated Cell Counter (Life Technologies) and CellTiter 96®AQueous One Solution (Promega) as specified by the manufacturer.

### Gene expression analysis

Total RNA from stimulated cells was extracted with the Nucleospin XS kit (Macherey Nagel, Germany). RNA was reverse-transcribed with the High-Capacity cDNA Archive Kit (Life Technologies, USA). The resulting cDNA was amplified using SYBR Green-based real-time PCR and the 7300 Real Time PCR system (Applied Biosystems, USA) following the manufacturer´s protocol. The specific primers are CGTCATCCATGGCGAACTG/GCTTCTTTGCAGCTCCTTCGT (*Actb*, a house-keeping gene coding for β-actin), AATCTATACCTGTCCTGTGTAATGAAAGAC/TGGGTATTGCTTGGGATCCA (*Il1b*), GTTCTCTGGGAAATCGTGGAAA/AAGTGCATCATCGTTGTTCATACA (*Il6*), GCAAAGAAACATGGACTTGAAGTTC/CACATGTCACTGCCCGAGAGT (*Il12b*), TGCTGGTCATTCCTGTCGT/TTGGTTTCTTGACCACCTTTTT (*Cd40*), ATGCTGCCCTGTCCTCCT/CCACAAGCCCAGTACGTGTAT (*Slc16a1*-MCT1), ATCGTGGGCACTCAGAAGTT/TTTGGTTGCATCCAGCAG (Slc16a3-MCT4) and GGCTGAGAAAAGCGGTATGA/TCGTTAACTCTCTCCGAGCTAGA (*Gpr81*). Relative mRNA levels (2^-ΔΔCt^) were determined by comparing (i) the cycle thresholds (Ct) for the gene of interest and *Actb* (ΔCt) and (ii) ΔCt values for treated and control groups (ΔΔCt). Ct upper limit was fixed to 33 cycles.

### Determination of cytokine production

IL-6 and IL-12 p40 concentration in cell-free supernatants were measured using ELISA kits (eBioscience, USA) following the manufacturer’s instructions.

### Cellular metabolic analysis

Seahorse XF24 Extracellular Flux Analyzer (Seahorse Biosciences) was used to obtain real-time measurements of glycolytic flux in macrophage measured by extracellular acidification rate (ECAR). Briefly, macrophages were seeded in 24-well Seahorse culture plates at a density of 150.000 cells/well. Before analysis of ECAR, growing medium was removed and cells were incubated in DMEM (Sigma) supplemented with glutamine (Invitrogen) (2mM). ECARs were analyzed using a cycle of 2 min of mixing followed by 3’ measurement cycle. Oligomycin (Sigma) and 2-deoxyglucose (Sigma) were injected at a final concentration of 2μM and 100mM respectively. ECARs were normalized to DNA content using Cyquant assay (Invitrogen). Data are presented as mean ± SEM.

### Statistical analysis

The Mann Whitney test was used for comparison between the two groups. For multiple comparisons, analysis of variance using Kruskall-Wallis test was performed. The differences were considered statistically significant for p < 0.05. All analyses were performed using the Graph Pad Prism program, version 5.0.

## Results & Discussion

**Fig 1:**
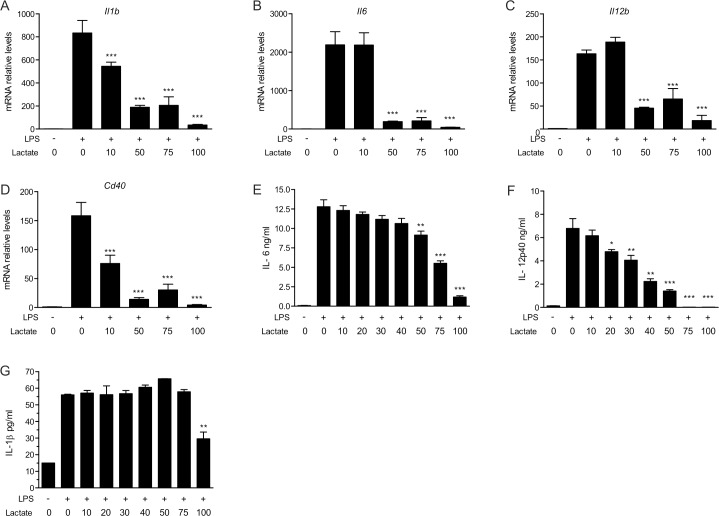
Lactate inhibits the LPS-mediated activation of bone marrow-derived macrophages. BMMs from C57BL/6 mice were incubated with various lactate concentrations in the presence or absence of LPS (100 ng/ml). (A-D) Messenger RNA levels were measured 3h post-stimulation and were expressed relative to those in mock cells that were not exposed to LPS or lactate (arbitrarily set to a value of 1). (E-G) Cytokine secretion by BMMs after 16h stimulation was measured by ELISA.The results are representative of four experiments and are expressed as the mean ± SEM. Statistically significant differences vs. LPS-treated cells are indicated as * p<0.05, ** p<0.01 and *** p<0.005.

**Fig 2:**
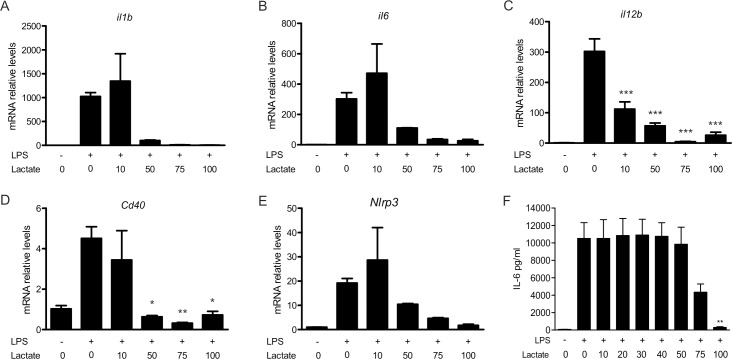
Lactate inhibits LPS-mediated activation of peritoneal macrophages. Peritoneal macrophages from C57BL/6 mice were incubated with various lactate concentrations in the presence or absence of LPS (100 ng/ml). (A-E) Gene expression was determined upon 3h of treatment. Messenger RNA levels were expressed relative to those in mock cells that were not exposed to LPS or lactate (arbitrarily set to a value of 1). (F) Il-6 secretion by peritoneal macrophages after 16h stimulation was measured by ELISA. The results are representative of two experiments and are expressed as the mean ± SEM. Statistically significant differences vs. LPS-treated cells are indicated as * p<0.05, ** p<0.01 and *** p<0.005.

To define the effect of lactate on the pro-inflammatory response of myeloid cells, we used TLR4-dependent activation by LPS of BMM (**Fig 1**). As expected, LPS stimulation increased transcription of *Il1b*, *Il6*, *Il12b*, and *Cd40* genes (**Fig 1A–1D**) and the secretion of IL-6, IL-12 p40 and IL-1b (**Fig 1E–1G**). In contrast, we observed that lactate inhibited the secretion and the transcription of these pro-inflammatory mediators in a dose-dependent manner. The experiments showed almost complete abrogation of activation at lactate concentration higher than 50 mM for most of the markers analyzed. In all conditions, using trypan blue and tetrazolium salt reduction, cell numbers were mostly unaffected and viability was shown >75% related to control cells (**S1 Fig**). A similar behavior was observed for peritoneal macrophages. Indeed, peritoneal macrophages showed high expression of pro-inflammatory markers *il1b*, *Il6*, *Il12b*, *Nlrp3* and *Cd40* upon LPS activation that was impaired by increasing concentrations of lactate (**Fig 2A–2E**). *Il12b* expression was found to be down-regulated at concentration of 10 mM lactate, whereas almost all other pro-inflammatory genes were no more expressed upon treatment with 50 mM lactate. As was observed for BMM, secretion of IL-6 induced by LPS was modulated by concentrations of lactate higher than 50 mM (Fig 2F). The present work thus corroborates the observations of Hoque *et al*. on the effect of lactate on wild type macrophages [16]. Importantly, treatment with lactate by itself did not alter the basal expression or secretion of the pro-inflammatory factors (**data not shown**). Lactate tissue levels may vary from 1 mM to 10 mM depending on tissue and metabolic state [19], and in pathological situation such as cancer these levels may be increased 2-to-5-fold [20–23]. Furthermore, some bacterial infections [8] may also rise tissue lactate levels from 10 to 30 mM. Consequently, our results indicate that pathophysiological levels of lactate, as reached *in vivo*, may have an impact on macrophage activation phenotype, as was also previously shown [10].

**Fig 3:**
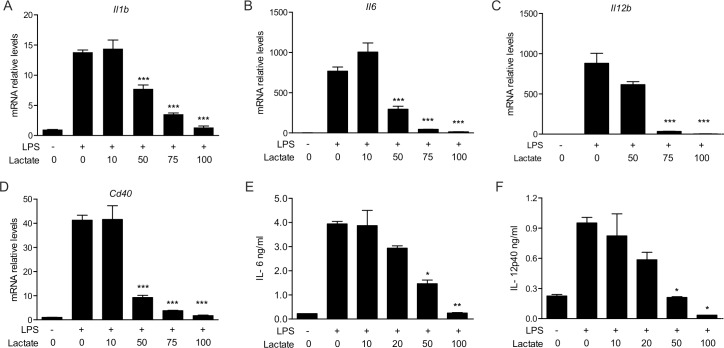
Lactate’s modulatory effects on LPS-stimulated BMMs are independent of GPR81. BMMs were derived from the bone marrow of *Gpr81*^*-/-*^and wild-type mice (littermate). The cells were incubated with various lactate concentrations in the presence or absence of LPS (100 ng/ml). (A-D) Gene expression was determined upon 3h of treatment. Messenger RNA levels were expressed relative to those in mock cells that were not exposed to LPS or lactate (arbitrarily set to a value of 1). (E-F) Cytokine secretion in *Gpr81*^*-/-*^ BMMs after 16h stimulation was measured by ELISA. The results are representative of two to four experiments and are expressed as the mean ± SEM. Statistically significant differences vs. LPS-treated cells are indicated as * p<0.05, ** p<0.01 and *** p<0.005.

GPR81, which is produced on adipocytes, was recently identified as the main lactate sensor in the regulation of fatty acid metabolism [14]. We further assessed GPR81’s contribution to lactate-mediated immunosuppression by studying cells derived from GPR81-deficient mice (*Gpr81*^*-/-*^) [17]. In contrast to Hoque *et al*. [16], we found that in murine *Gpr81*^*-/-*^ BMMs transcription of the pro-inflammatory genes *Il1b*, *Il6*, *Il12b*, and *Cd40* was down-modulated by lactate treatment (**Fig 3A–3D**). Furthermore,. these cells responded to lactate by downregulating the LPS-mediated IL-6 and IL-12 p40 secretion, following a dose response similar to that observed for GPR81-proficient cells (**Fig 3E and 3F**). As previously shown [14], *Gpr81* was highly expressed in visceral or subcutaneous fat tissue but the expression was 100-fold lower in secondary lymphoid organs or gut tissues (**S2A Fig**). Furthermore, *Gpr81*m RNA levels in BMMs were >1000-fold lower than those in adipose tissue (**S2B Fig**). Taken together, these data indicate that lactate can induce modulation of the pro-inflammatory response via a GPR81-independent signaling pathway. Interestingly, we observed that peritoneal macrophages also used in the Hoque *et al*.’s study, displayed higher levels of *Gpr81* expression than BMMs (**S2B Fig**). However, activation of GPR81-deficient peritoneal macrophages was also inhibited by increasing concentrations of lactate (**S3 Fig**). These data suggest that (1) *Gpr81* expression and GPR81 activity may be environmentally controlled in macrophages, and (2) macrophages can respond to lactate in specific conditions in a GPR81-independent manner.

**Fig 4:**
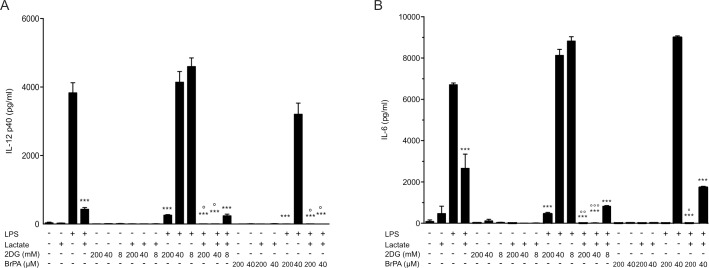
Inhibition of glycolysis impairs expression of pro-inflammatory mediators by macrophages. BMMs from C57BL/6 mice were incubated for 16h with 75 mM lactate±LPS (100 ng/ml) ± glycolysis inhibitors (2-deoxyglucose: 2DG; 3-Bromopyruvate: Br-PA) at indicated concentrations. Cytokine secretion by BMMs was measured by ELISA for IL-12 p40 (A) and IL-6 (B). The results are representative of two experiments and are expressed as the mean ± SEM. Statistically significant differences vs. LPS-treated cells are indicated as * p<0.05, ** p<0.01 and *** p<0.005; whereas differences vs. LPS+lactate treated cells are indicated as ° p<0.05, °° p<0.01 and °°° p<0.005.

**Fig 5:**
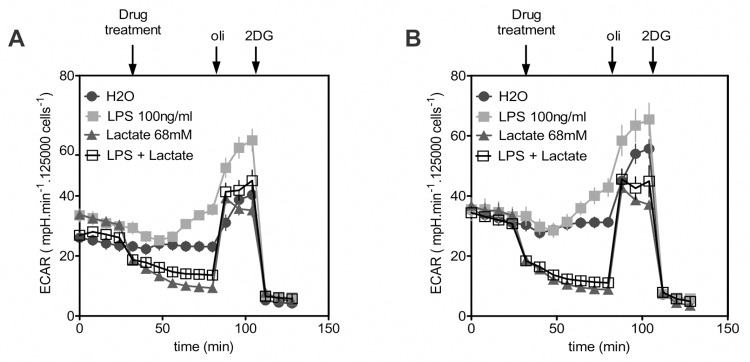
Extracellular lactate abrogates LPS-induced macrophage glycolysis in a GPR81-independent manner. The extracellular acidification rate (ECAR) was measured as a surrogate marker of glycolysis using a SeaHorse Extracellular Flux analyzer. The cells were initially stimulated with LPS (100 ng/ml) and/or lactate (68 mM), and water alone was used as an additive for a non-stimulated control condition. In all the experiments, sequential treatment with oligomycin (oli) and then 2-deoxyglucose (2DG) was used to inhibit the mitochondrial respiratory chain and block glycolysis, respectively. Similar experiments were performed with C57BL/6 (A) and *Gpr81*^*-/-*^ BMMs (B). The results are representative of three experiments and are expressed as the mean ± SEM.

Glycolysis is essential for the production of pro-inflammatory response by macrophages and dendritic cells [13] and extensive metabolic changes usually occur upon macrophage activation. Particularly, pro-inflammatory signals promote a rise in the glycolysis rate, inhibition of oxygen consumption, and increased mitochondria use of aspartate-argininosuccinate shunt to replenish the oxaloacetate pool [24, 25]. These changes facilitate generation of NO and lipid mediators of inflammation such as specific eicosanoids [24, 26]. It has been shown that perturbation of this metabolic remodeling by glycolysis inhibition abrogates IL6 production by macrophages upon LPS stimulation [26]. In agreement with these results, we observed that inhibitors of glycolysis abrogate LPS-induced production of pro-inflammatory mediators in BMM to similar extent of lactate (**Fig 4**). Both 2-deoxyglucose and 3-bromopyruvate inhibited the first steps of glycolysis (**S4 Fig**). Using these inhibitors we observed a decrease in the production of IL-6 and IL-12p40 in a dose dependent manner. Furthermore, the effects of the inhibitor were reinforced in the presence of lactate 75 mM, showing an additive effect (**Fig 4**). These results suggest that lactate may contribute to modulation of glycolysis activity upon LPS activation. In order to further determine the impact of lactate on macrophage metabolism, we measured the extracellular acidification rate (ECAR, a surrogate marker of glycolytic activity). As previously reported [27], LPS stimulated the ECAR, whereas lactate was found to fully abrogate the LPS-mediated induction of glycolysis (**Fig 5A**). Interestingly, *Gpr81*^*-/-*^and wild-type BMMs responded similarly to lactate and LPS (**Fig 5B**); in both cases, treatment with lactate fully abrogated the rise in the glycolysis rate upon LPS stimulation. In both cell genotypes, the treatment with lactate in the absence of LPS also resulted in a lower rate of glycolysis. The monocarboxylate transporters MCT4 and MCT1 could be the major transporters responsible for lactate secretion or influx, respectively [28]. It was recently shown that the lactate membrane transporter MCT4 is induced in macrophages upon TLR activation, being critical for the transport of lactate produced upon cell activation [27]. Blockade of the macrophages’ capacity to export lactate [27] or high concentrations of lactate in the extracellular milieu [29] have been shown to dramatically decrease the glycolysis rate, even upon LPS activation. We evaluated MCT4 and MCT1 expression in macrophages used for our experiments (**S5 Fig**). In all cases, both genes were expressed resulting in Cts for amplification around 25 cycles (not shown), indicating intermediate levels of expression. BMM showed increased expression of MCT1 and MCT4 upon lactate incubation, whereas basal levels were not significantly changed in lactate-treated peritoneal macrophages. These results indicate that lactate can be traslocated from outside or inside following gradient concentrations in the conditions used in our experiments. Consequently, a raise in extracellular lactate can be mirrored by a raise in intracellular lactate facilitated by MCT action, and this may then influence in other cellular activities such as glycolisis, as previously described [30]. A correlation between the blockade of glycolysis activity and the lack of macrophage pro-inflammatory activation upon LPS stimulation was observed [27, 29]. Interestingly, it was recently shown by *in vitro* and *in vivo* approaches that impaired glycolytic activity is a hallmark of the “immunoparalysis” phenotype observed in circulating leukocytes upon severe sepsis corresponding to a state of low cytokine production upon proinflammatory stimulation [31]. The GPR81-independent modulatory effects on pro-inflammatory activation described herein may also be associated to such modulation of metabolic pathways in myeloid cells.

There is an increasingly large body of evidence showing that lactate can modulate immune cell function [5, 10, 32]. Local lactate concentrations in different tissues can reach the values that were modulatory in our model system [5, 32]. Thus lactate can have functional consequences as shown in tumor models [10]. It is known that tumor progression is correlated with the production of lactate [33], pointing to the importance of immunopathological mechanisms triggered by this metabolite. Lactate’s modulatory effects on cell types that are critical players in the orchestration of the immune response may contribute significantly to this observation. Results shown here contribute to the elucidation of the mechanisms responsible for lactate bioactivity, which might facilitate the development of new therapeutic strategies for critical health problems such as inflammatory bowel disease and cancer.

## Supporting Information

S1 Fig High concentrations of lactate do not significantly impair macrophage viability. Bone marrow-derived macrophages (BMM, A and C) or peritoneal macrophages (PM, B and D) from C57BL/6 mice were incubated for 16h with various lactate concentrations in the presence or absence of LPS (100 ng/ml). (A-B) Viable cell numbers were measured using trypan blue staining and an automated cell counter Countess^TM^. (C-D) Macrophage viability was also assessed using a tetrazolium salt reduction assay and measurement of absorbance at 490 nm (CellTiter 96® AQueous One Solution). Total absence of viability was associated with an absorbance at 490 nm of 0.164 ± 0.006. The results are representative of two experiments and are expressed as the mean ± SEM. Statistically significant differences vs. LPS-treated cells are indicated as * p<0.05, ** p<0.01 and *** p<0.005.
(TIF)

S2 Fig *Gpr81* transcript levels are very low in macrophages. *Gpr81* mRNA levels were assessed by real time quantitative RT-PCR in (A) different tissues from C57BL/6 mice, and in (B) peritoneal macrophages (PM) and bone marrow-derived macrophages (BMM) from C57BL/6 mice. Messenger RNA levels are expressed relative to those in visceral fat tissue (arbitrarily set to a value of 1). The results are representative of two experiments and are expressed as the mean ± SEM. Statistically significant differences vs. visceral fat are indicated as * p<0.05, ** p<0.01 and *** p<0.005.
(TIF)

S3 Fig Lactate inhibits LPS-mediated activation of *Gpr81*^*-/-*^ peritoneal macrophages. Peritoneal macrophages from C57BL/6 and *Gpr81*^*-/-*^ mice were incubated for 16h with various lactate concentrations in the presence or absence of LPS (100 ng/ml). Cytokine secretion was measured by ELISA. The results are representative of two experiments and are expressed as the mean ± SEM. Statistically significant differences vs. LPS-treated cells are indicated as * p<0.05, ** p<0.01 and *** p<0.005.
(TIF)

S4 Fig Molecular targets of different metabolic inhibitors. Schematic representation of central carbon metabolism. The steps that are inhibited by the different compounds used in Fig 4 are indicated.
(TIF)

S5 Fig Lactate transportesrs MCT1 and MCT4 are expressed in macrophages. MCT1 (*Slc16a1*) and MCT4 (*Slc16a4)* mRNA levels were assessed by real time quantitative RT-PCR in BMMs (A-B) and peritoneal macrophages (C-D) used for our studies. Messenger RNA levels in treated cells are expressed relative to those in untreated (arbitrarily set to a value of 1). The results are representative of two experiments and are expressed as the mean ± SEM.
(TIF)
