# Supplementary figures and images for: Lactate Inhibits the Pro-Inflammatory Response and Metabolic Reprogramming in Murine Macrophages in a GPR81-Independent Manner
Source: PLoS One. 2016 Nov 15;11(11):e0163694. doi: 10.1371/journal.pone.0163694 (PMC5112849; doi:10.1371/journal.pone.0163694)

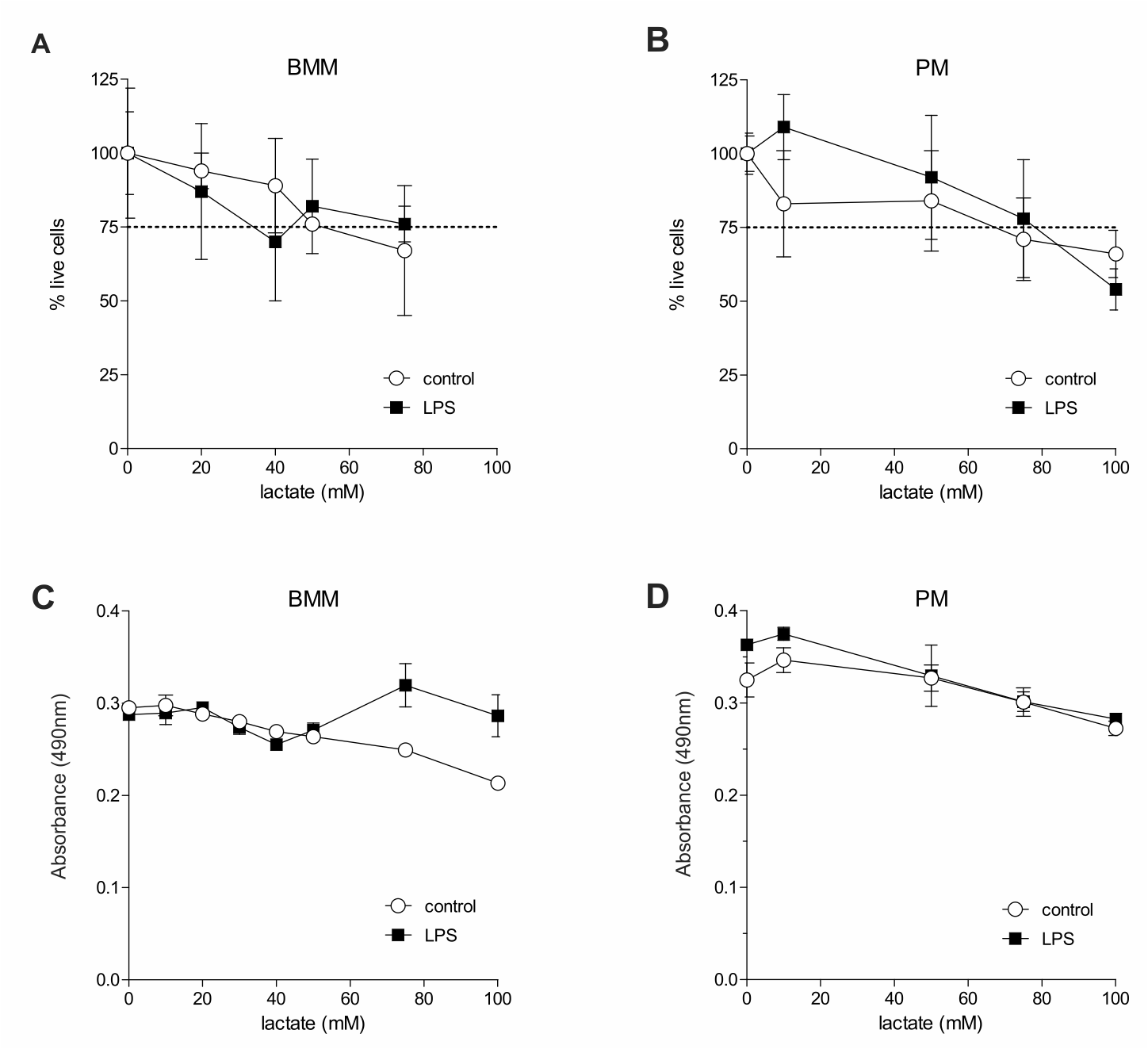

Supplement: S1 Fig — Bone marrow-derived macrophages (BMM, A and C) or peritoneal macrophages (PM, B and D) from C57BL/6 mice were incubated for 16h with various lactate concentrations in the presence or absence of LPS (100 ng/ml). (A-B) Viable cell numbers were measured using trypan blue staining and an automated cell counter CountessTM. (C-D) Macrophage viability was also assessed using a tetrazolium salt reduction assay and measurement of absorbance at 490 nm (CellTiter 96® AQueous One Solution). Total absence of viability was associated with an absorbance at 490 nm of 0.164 ± 0.006. The results are representative of two experiments and are expressed as the mean ± SEM. Statistically significant differences vs. LPS-treated cells are indicated as * p<0.05, ** p<0.01 and *** p<0.005. (TIF) [file pone.0163694.s001.tif]

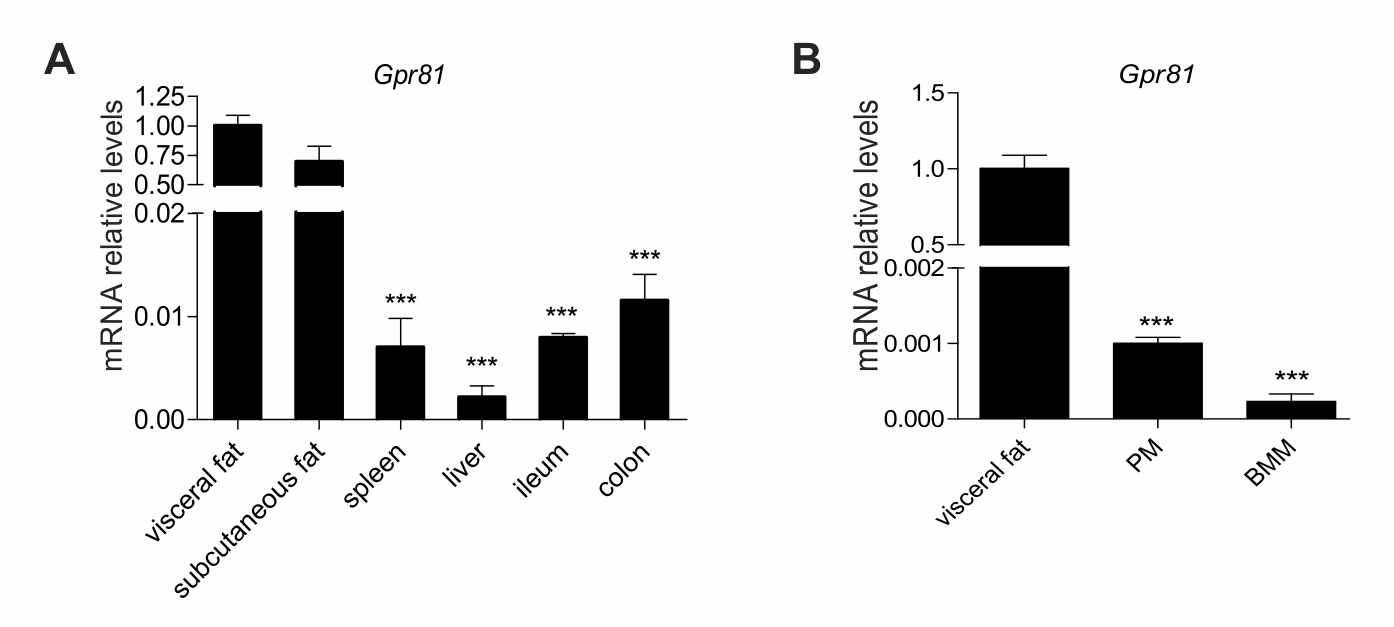

Supplement: S2 Fig — Gpr81 mRNA levels were assessed by real time quantitative RT-PCR in (A) different tissues from C57BL/6 mice, and in (B) peritoneal macrophages (PM) and bone marrow-derived macrophages (BMM) from C57BL/6 mice. Messenger RNA levels are expressed relative to those in visceral fat tissue (arbitrarily set to a value of 1). The results are representative of two experiments and are expressed as the mean ± SEM. Statistically significant differences vs. visceral fat are indicated as * p<0.05, ** p<0.01 and *** p<0.005. (TIF) [file pone.0163694.s002.tif]

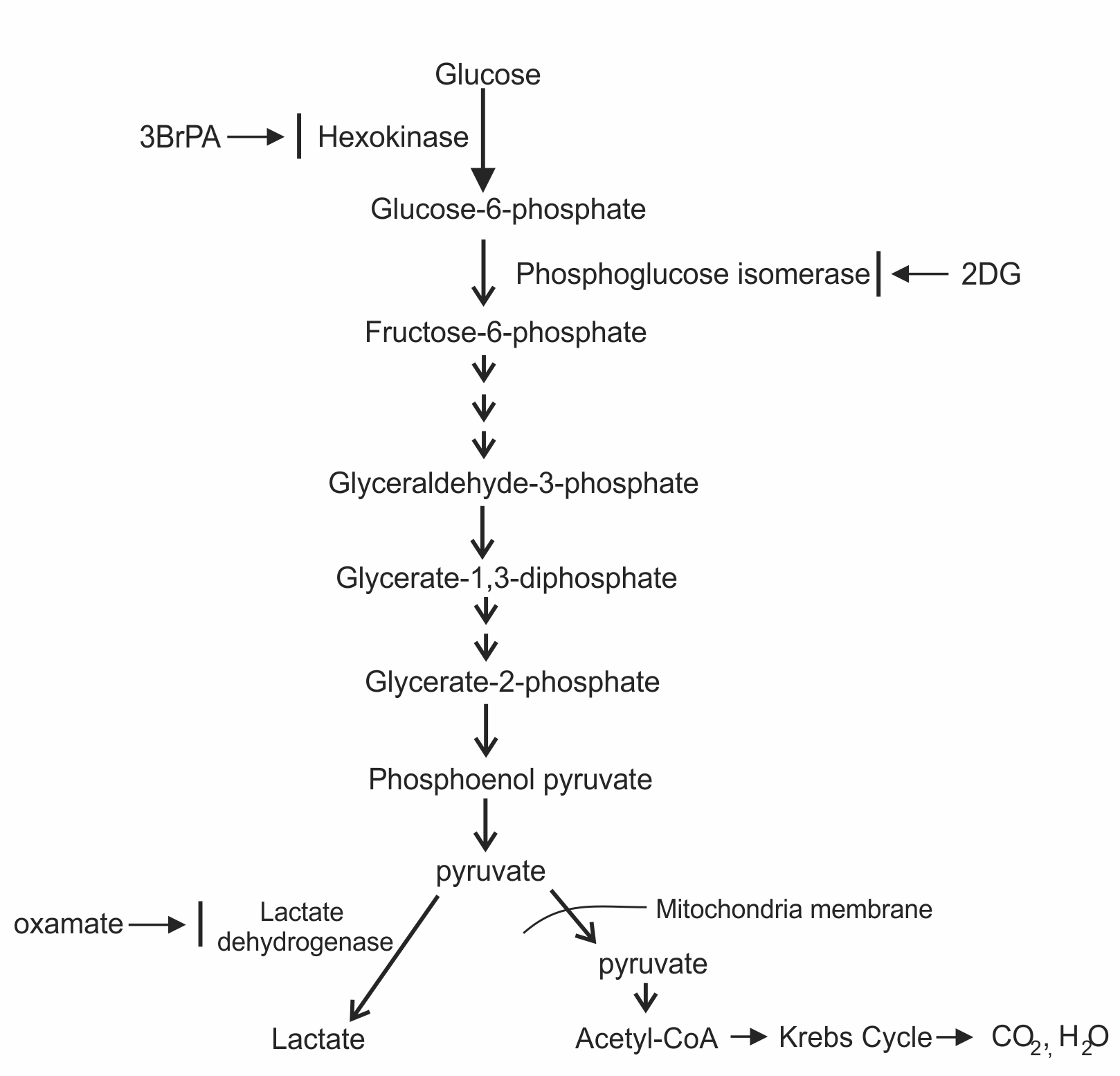

Supplement: S3 Fig — Peritoneal macrophages from C57BL/6 and Gpr81-/- mice were incubated for 16h with various lactate concentrations in the presence or absence of LPS (100 ng/ml). Cytokine secretion was measured by ELISA. The results are representative of two experiments and are expressed as the mean ± SEM. Statistically significant differences vs. LPS-treated cells are indicated as * p<0.05, ** p<0.01 and *** p<0.005. (TIF) [file pone.0163694.s003.tif]

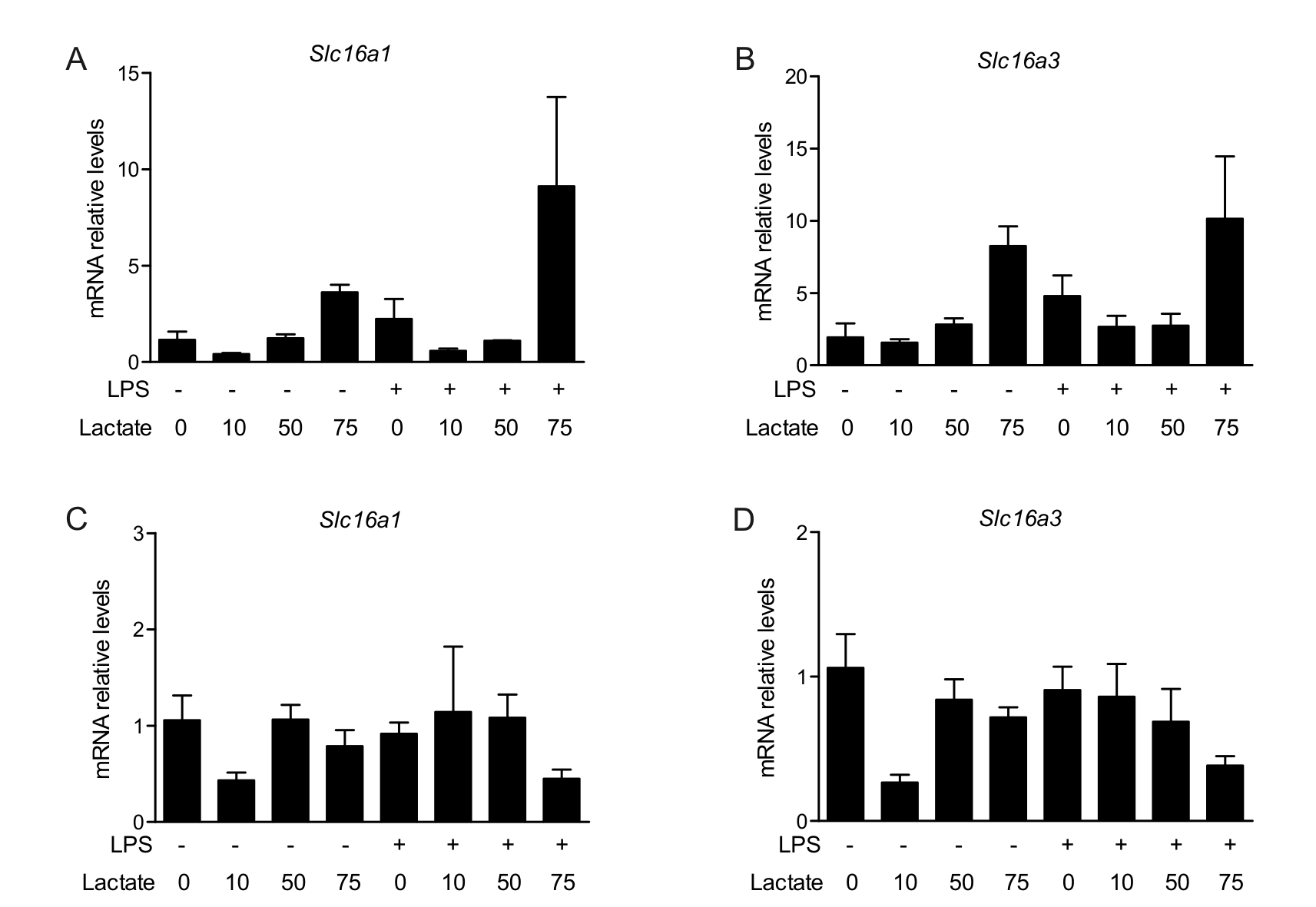

Supplement: S5 Fig — MCT1 (Slc16a1) and MCT4 (Slc16a4) mRNA levels were assessed by real time quantitative RT-PCR in BMMs (A-B) and peritoneal macrophages (C-D) used for our studies. Messenger RNA levels in treated cells are expressed relative to those in untreated (arbitrarily set to a value of 1). The results are representative of two experiments and are expressed as the mean ± SEM. (TIF) [file pone.0163694.s005.tif]
